# Spleen tyrosine kinase facilitates neutrophil activation and worsens long-term neurologic deficits after spinal cord injury

**DOI:** 10.1186/s12974-021-02353-2

**Published:** 2021-12-24

**Authors:** Dylan A. McCreedy, Clare L. Abram, Yongmei Hu, Sun Won Min, Madison E. Platt, Megan A. Kirchhoff, Shelby K. Reid, Frank L. Jalufka, Clifford A. Lowell

**Affiliations:** 1 Department of Biology, Texas A&M University, 301 Old Main Dr, ILSB 3128, College Station, TX 77843 USA; 2 Texas A&M Institute for Neuroscience, Texas A&M University, College Station, TX 77843 USA; 3 Department of Laboratory Medicine and Immunology Program, University of California, San Francisco, CA 94143 USA

**Keywords:** Spinal cord injury, Neutrophils, Inflammation, Apoptosis, Cytokines, Degranulation, Neutrophil extracellular traps

## Abstract

**Background:**

Spinal cord injury elicits widespread inflammation that can exacerbate long-term neurologic deficits. Neutrophils are the most abundant immune cell type to invade the spinal cord in the early acute phase after injury, however, their role in secondary pathogenesis and functional recovery remains unclear. We have previously shown that neutrophil functional responses during inflammation are augmented by spleen tyrosine kinase, Syk, a prominent intracellular signaling enzyme. In this study, we evaluated the contribution of Syk towards neutrophil function and long-term neurologic deficits after spinal cord injury.

**Methods:**

Contusive spinal cord injury was performed at thoracic vertebra level 9 in mice with conditional deletion of Syk in neutrophils (Syk^f/f^MRP8-Cre). Hindlimb locomotor recovery was evaluated using an open-field test for 35 days following spinal cord injury. Long-term white matter sparing was assessed using eriochrome cyanide staining. Blood-spinal cord barrier disruption was evaluated by immunoblotting. Neutrophil infiltration, activation, effector functions, and cell death were determined by flow cytometry. Cytokine and chemokine expression in neutrophils was assessed using a gene array.

**Results:**

Syk deficiency in neutrophils improved long-term functional recovery after spinal cord injury, but did not promote long-term white matter sparing. Neutrophil activation, cytokine expression, and cell death in the acutely injured spinal cord were attenuated by the genetic loss of Syk while neutrophil infiltration and effector functions were not affected. Acute blood-spinal cord barrier disruption was also unaffected by Syk deficiency in neutrophils.

**Conclusions:**

Syk facilitates specific neutrophil functional responses to spinal cord injury including activation, cytokine expression, and cell death. Long-term neurologic deficits are exacerbated by Syk signaling in neutrophils independent of acute blood-spinal cord barrier disruption and long-term white matter sparing. These findings implicate Syk in pathogenic neutrophil activities that worsen long-term functional recovery after spinal cord injury.

## Background

Spinal cord injury (SCI) results in extensive and persistent inflammation that can exacerbate secondary tissue damage and worsen long-term neurological outcomes. Neutrophils are the most common circulating leukocyte subtype in human blood and the first to invade the injured spinal cord in large numbers [1–3]. High abundance of circulating neutrophils early after injury has recently been found to correspond with lower likelihood of functional recovery in individuals with SCI [4–6]. In murine models of SCI, greater neutrophil recruitment from the blood into the acutely injured spinal cord has commonly been associated with increased secondary tissue damage and worsening of neurologic deficits [4, 7–10]. However, antibody-mediated depletion of neutrophils after SCI has produced variable effects on long-term recovery [4, 11–14], potentially due to removal of diverse subsets or functions of neutrophils that differentially contribute to tissue damage and repair. The mechanisms by which neutrophils contribute to secondary pathogenesis and neurologic deficits after SCI currently remain unclear.

Neutrophils have a diverse array of cellular responses that enable context-dependent activation of pro- and anti-inflammatory functions [1, 15]. Activated neutrophils can trigger a variety of cytotoxic effector functions including the production of reactive oxygen and nitrogen species (ROS/RNS), formation of neutrophil extracellular traps (NETs) and degranulation [16–21]. While imperative for sterilizing sites of injury, neutrophil effector functions can also result in collateral damage to adjacent tissue and amplify pathogenic inflammatory responses. Activated neutrophils can also undergo apoptosis at the injury or infection site and are normally phagocytosed and cleared by macrophages. The phagocytosis of apoptotic neutrophils by macrophages has been shown to induce an anti-inflammatory phenotype in the macrophages, which may promote tissue repair and resolution of inflammation [22–25]. However, this process may be impaired in the injured spinal cord where macrophages extensively phagocytose myelin debris and lose their capacity to clear dead neutrophils [26, 27]. The cytotoxic cellular contents of dead neutrophils may then leak in the tissue parenchyma and cause unintended damage. In other models of inflammation, neutrophils have also been shown to produce cytokines that influence inflammation and wound healing [28–31]. Little is known, however, about the diverse functions utilized by neutrophils in the injured spinal cord or the signaling pathways that mediate pathogenic neutrophil responses to SCI.

To enable rapid and specific responses to inflammatory stimuli present at the site of injury or infection, neutrophils express a wide repertoire of immunoreceptors including FcγRs and integrins [32–34]. Spleen tyrosine kinase (Syk) is a prominent signaling mediator in neutrophils that interacts with immunoreceptor tyrosine activation motifs (ITAMs) to help coordinate intracellular responses to the collective stimuli encountered during inflammation [33, 35–37]. Syk can contribute to the development of neutrophil effector functions, as well as cytokine secretion, to facilitate the clearance of microbial infection [38, 39]. While beneficial for fighting pathogenic microbes, Syk has also been found to exacerbate destructive neutrophil responses and tissue damage in multiple inflammatory conditions including arthritis, thrombohemorrhagic vasculitis, epidermolysis bullosa acquisita, and asthma [40–45]. Syk signaling, therefore, may play a critical role in neutrophil responses and functional recovery after SCI.

To determine if Syk contributes to pathogenic neutrophil activities in the injured spinal cord, as well as long-term neurologic deficits, we examined a neutrophil-specific knockout of Syk in a murine thoracic contusion model of SCI. Mice expressing Cre recombinase under control of the MRP8 gene regulator elements (MRP8-Cre) were bred with Syk^f/f^ mice to produce conditional deletion of Syk in neutrophils. Following SCI, neutrophil-specific deletion of Syk led to improved long-term functional recovery, but did not reduce long-term white matter tissue loss or acute blood-spinal cord barrier disruption. Syk deficiency attenuated neutrophil activation, cytokine expression, and cell death, but did not alter neutrophil accumulation or effector functions acutely after SCI. Collectively, these findings demonstrates that Syk signaling influences neutrophil phenotype and function in the acutely injured spinal cord and exacerbates long-term neurologic deficits after SCI.

## Methods

### Syk^f/f^MRP8Cre mice

All studies were performed in accordance with protocols approved by the Institutional Animal Care and Use Committee at the University of California, San Francisco. Syk^f/f^ and MRP8-Cre (The Jackson Laboratory) mice were independently maintained on a C57Bl/6 background and bred to generate Syk^f/f^ and Syk^f/f^MRP8-Cre littermate mice for this study, as previously described [38, 40]. Adult male and female mice (~ 3 to 5 months old) were used in all experiments. Mice were housed in groups of two to five prior to injury and female (F) mice were housed in groups of two to three after SCI, whereas males (M) were singly housed.

### Spinal cord injury

Spinal cord contusion injury was performed as previously described [46]. Briefly, mice were anesthetized with 2% isoflurane, a laminectomy was performed at thoracic vertebrate level 9 (T9), and a 2 g weight was dropped 7.5 cm onto the exposed dura mater to produce a moderate contusion SCI [47]. Following the SCI, the cut musculature was sutured together, and the skin incision was closed with wound clips. Mice also received a topical administration of bupivacaine at the incision site and a subcutaneous injection of saline. A circulating water pad set at 37 °C was used to maintain body temperature during and after surgery. For 10 days following SCI, mice received daily subcutaneous injections of saline and antibiotic (enrofloxacin, 2.5 mg/kg). Manual bladder expression was performed twice daily until euthanasia.

### Experimental design

The primary goal of this study was to determine the contribution of Syk signaling on neutrophil phenotype and effector functions in the acutely injured spinal cord, as well as long-term functional recovery after SCI. We utilized MRP8-Cre and Syk^f/f^ mice to obtain conditional deletion of Syk in neutrophils as previously described [38, 40]. Syk^f/f^MRP8-Cre mice lack Syk kinase in neutrophils but continue to express normal levels of Syk in other myeloid and B-cell leukocyte subsets. The deficiency of Syk kinase in neutrophils does not alter granulopoiesis; Syk^f/f^MRP8-Cre mice have normal neutrophil numbers in the peripheral blood, spleen and bone marrow. Male and female littermate adult mice were used throughout the study. Sex-specific effects were evaluated for experiments with a sample size of three or more for each sex and in which a significant difference was detected between genotypes. Time points for data collection and sample sizes were determined based on previous studies and experience, as well as power analysis. SCI surgeries and behavioral analysis were performed with the researchers blinded to the genotype. Block randomization was used for all experiments.

### Open field locomotor testing

To assess functional recovery, open field locomotor testing using the Basso Mouse Scale (BMS) was performed at 1, 3, and 7 days post-SCI and then weekly thereafter until euthanasia at five weeks post-SCI. The BMS test assesses multiple aspects of hindlimb movement, paw placement, stepping and coordination [48]. Mice were acclimated to an open field arena (53 × 108 × 5.5 cm) in 4 min sessions on 3 consecutive days during the week prior to injury. After SCI, mice were placed into the open field and scored using the 9-point BMS during a 4-min testing period by observers blinded to the genotype.

### White matter sparing

Animals were euthanized at 35 days post-SCI and transcardially perfused with 35 mL of PBS followed by 35 mL of 4% paraformaldehyde in PBS. The spinal cords (4 mm centered over the injury site) were dissected and post-fixed overnight at 4 °C. Spinal cords were then cryoprotected in 30% sucrose, embedded, and frozen at − 80 °C. 20 µm transverse cryosections spanning the lesion site were obtained and every tenth section was selected for eriochrome cyanine staining. Images were captured using a Leica DM 6B microscope equipped with a × 10 objective (0.32 NA) and a Leica DM45000 color camera. The total area of residual myelin with was hand-traced and quantified using ImageJ. The section with the least total myelin area was selected as the lesion epicenter.

### Neutrophil counting

Animals were euthanized at 1d post-SCI and 25 µm transverse cryosections were obtained as described above for “White matter sparing”. Every twentieth section (~ 500 µm between sections) was labeled with AlexaFluor647-Ly6G (Clone 1A8, 1:200 dilution, Biolegend), anti-NeuN (1:1000, Millipore), and FluoroMyelin Green (1:300, ThermoFisher Scientific). Sections were then washed and incubated with an AlexaFluor 555-conjugated secondary antibody (1:500, Life Technologies). Images were captured using a Nikon Eclipse upright microscope equipped with a × 10 objective (0.45 NA). Neutrophils were counted using custom Matlab code with regions of interest (ROIs) manually drawn for spinal cord grey matter, white matter, and meninges for each section. The section with the greatest number of neutrophils was designated as the lesion epicenter.

### Flow cytometry analysis

Mice were anesthetized by i.p. injection of 2.5% avertin (0.02 mL/g body weight; Sigma) and peripheral blood was obtained by cardiac puncture using a heparin-coated syringe. Approximately 5 mm of spinal cord tissue centered on the injury site was rapidly dissected and placed into RPMI media on ice. The spinal cord tissue was mechanically dissociated using a plastic tissue pestle and filtered through a 100 µm nylon mesh cell strainer. RBC lysis (Pharm Lyse, BD) was performed in all blood and spinal cord samples for 5 min on ice and quenched with FACS buffer (0.5 µM EDTA and 2% heat-inactivated fetal bovine serum in HBSS). Following centrifugation, the cell pellet was washed with HBSS and live-dead cell staining was performed using Zombie Dye Red (1:500 dilution, Biolegend) for 30 min at 4 °C. Samples were washed with FACS buffer and Fc blocking was performed using an anti-mouse CD16/32 antibody (1:100 dilution, Clone 93, eBioscience) for 20 min at 4 °C. Samples were washed with FACS buffer and partitioned for surface and intracellular staining.

For surface staining, samples were incubated with a cocktail of the following antibodies in FACS buffer for 30 min at 4 °C: APC/Cy7-CD45 (Clone 30-F11, 1:200 dilution, Biolegend), APC-CD11b (Clone M1/70, 1:200 dilution, Biolegend), Pacific Blue-Ly6G (Clone 1A8, 1:200 dilution, Biolegend), PerCP/Cy5.5-Ly6C (Clone HK1.4, 1:600 dilution, Biolegend), and FITC-CD62L (Clone Mel-14, 1:100 dilution, Biolegend). Following incubation, samples were washed twice with FACS buffer and counting beads (AccuCheck, ThermoFisher Scientific) were added to the spinal cord samples to determine total cell number.

For ROS labeling, dissociated spinal cord and heparinized blood samples were incubated with DHR123 (1:5000, ThermoFisher Scientific) for 30 min at 37 °C. The cells were then washed with cold RPMI media and centrifuged at 400 g for 5 min at 4 °C. RBC lysis and Fc blocking was performed as described above and samples were incubated with a cocktail of the following antibodies in FACS buffer for 30 min at 4 °C: APC/Cy7-CD45, APC-CD11b, Pacific Blue-Ly6G and PerCP/Cy5.5-Ly6C.

For intracellular staining, samples were first incubated with the following cocktail of antibodies in FACS buffer for 30 min at 4 °C: APC/Cy7-CD11b, Pacific Blue-Ly6G, and PerCP/Cy5.5-Ly6C. Samples were then washed twice with FACS buffer and fixed using the Cytofix/Cytoperm kit (BD) according to the manufacturer’s protocol. After fixation, samples were washed twice with permeabilization buffer and incubated with one of the following primary antibodies in permeabilization buffer for 30 min at 4 °C: AlexaFluor 647-conjugated anti-Syk (1:500, UCSF mAb Core), anti-MRP8 (1:2000, R&D Systems), or anti-CitH3 (1:2000, Abcam). Samples were washed twice with NDS-FACS buffer (HBSS containing 2% normal donkey serum (NDS) and 0.5 µM EDTA) and incubated with an AlexaFluor 488-conjugated secondary antibody (1:500, Life Technologies) in NDS-FACS buffer for 30 min at 4 °C. Samples were washed twice with NDS-FACS buffer and resuspended for flow cytometry analysis.

Flow cytometry was performed using a Fortessa flow cytometer (BD). Negative and positive fluorescent signals for compensation were obtained using the anti-rat/hamster and amine reactive compensation bead kits (ThermoFisher Scientific). Flow cytometry data was analyzed and geometric mean fluorescent intensity (G-MFI) values were calculated using FlowJo software (BD). Doublet cells were removed using FSC-H by FSC-A gating. Isotype control (for antibody labeling) or negative staining (for ROS labeling) groups were used to establish gates and to determine background G-MFI values that were subtracted from the reported G-FMI values.

### Immunoblotting

Spinal cord tissue (5 mm centered over the injury site) was rapidly dissected and homogenized in RIPA buffer (ThermoFisher Scientific) supplemented with a protease inhibitor cocktail (Roche) on ice. Samples were centrifuged to remove insoluble debris and protein concentration in the homogenates was determined using a BCA protein assay kit (Pierce). For each sample, 20 µg of protein was loaded into a 4–12% Bis–Tris gel (ThermoFisher Scientific) and transferred onto a PVDF membrane. The membrane was blocked with 5% bovine serum albumin (BSA) in Tris-buffered saline (TBS) for 2 h and then incubated for 1 h at 4 °C with donkey anti-mouse IgG IRDye 800 (1:1000 dilution, LI-COR) in TBS containing 0.1% triton (TBST) and 5% BSA. The membrane was washed three times with TBST and imaged using an Odyssey CLx imager (LI-COR). GAPDH (Millipore) was used as an internal loading control. The bands at ~ 50 kDa and ~ 25 kDa corresponding to the IgG heavy and light chain subunits, respectively, were quantified and normalized to GAPDH using Image Studio Lite software (LI-COR).

### Gene expression array

Neutrophils were isolated from the blood and spinal cords (5 mm centered over the injury site) of Syk^f/f^ and Syk^f/f^MRP8-Cre mice at 1 days post-SCI using magnetic bead separation. Neutrophils from the blood from Syk^f/f^ uninjured mice were also isolated as a control population. Blood and spinal cord samples were collected and dissociated as described above for “Flow cytometry analysis”. Following RBC lysis and Fc blocking, cells were incubated with PE-Ly6G antibody (Clone 1A8, 1:100 dilution, Biolegend) for 20 min at 4 °C. Cells were washed and then incubated with magnetic UltraPure Anti-PE MicroBeads (1:5 dilution, Miltenyi) for 20 min at 4 °C. Cells were washed again and labeled neutrophils were isolated using LS columns (Miltenyi) and a QuadroMACS Separator (Miltenyi) according to the manufacturer’s protocol. Magnetic isolation was then performed again to further increase the purity of the isolated neutrophil population. All buffers and reagents were kept on ice or at 4 °C during the isolation to reduce transcriptional changes in the isolated cell population.

Isolated neutrophils from the blood samples were counted and ~ 2 × 10^4^ purified neutrophils from the blood and ~ 4/5 of the purified neutrophils from the spinal cord were lysed in TRI Reagent (Zymo). The remaining cells were used for flow cytometry analysis to determine neutrophil purity. RNA purification was performed using a Direct-zol RNA Microprep kit (Zymo) according to manufacterur’s instructions. Reverse transcription of RNA into cDNA was performed using SuperScript IV VILO (ThermoFisher Scientific). The resulting cDNA was stored at − 80 °C until further use.

Gene expression analysis of 40 genes was performed using the Biomark 48.48 Dynamic Array (Fluidigm) according to the manufacturer’s protocol. Primers (Table 1, IDT or RealTimePrimers.com) were previously validated for specificity using the NIH BLAST search and tested for linearity using serially diluted cDNA from pooled blood and spinal cord neutrophil samples. Briefly, all primers were pooled and pre-amplification of each cDNA sample was performed for 10 cycles using a thermocycler. Primers and pre-amplified samples were then loaded into the gene expression array and analyzed on a Biomark HD system (Fluidigm). Delta (Δ) CT values for each gene were determined by subtracting the average CT value for three housekeeping genes (*Gapdh*, *Actb*, and *Hprt1*). Genes in which CT values could not be obtained for all or more than half of the biological replicates were considered not detected (n.d.).

**Table 1:** Primer sequences for gene array

Gene symbol	Forward primer (5′–3′)	Reverse primer (5′–3′)	Accession number
Actb	AGGGAAATCGTGCGTGACAT	GAACCGCTCGTTGCCAATAG	NM_007393.5
Angpt1	GGGGGAGGTTGGACAGTAA	CATCAGCTCAATCCTCAGC	NM_009640.4
Arg1	CTCCAAGCCAAAGTCCTTAGAG	AGGAGCTGTCATTAGGGACATC	NM_007482.3
Bcl2a1	GATACGGCAGAATGGAGGTT	GCATTTCCCAGATCTGTCCT	NM_009742.3
C1qa	CAAGGACTGAAGGGCGTGAA	CAAGCGTCATTGGGTTCTGC	NM_007572.2
C3	CACCGCCAAGAATCGCTAC	GATCAGGTGTTTCAGCCGC	NM_009778.3
Ccl2	TGCTACTCATTCACCAGCAA	GTCTGGACCCATTCCTTCTT	NM_011333.3
Ccl3	GACAAGCTCACCCTCTGTCA	GCCGGTTTCTCTTAGTCAGG	NM_011337.2
Ccl4	AGCAACACCATGAAGCTCTG	CTGTCTGCCTCTTTTGGTCA	NM_013652.2
Ccl9	AGTGGTCTGTGGGACTTTGG	CAGACCTGTGGCTGCATAGA	NM_011338.2
Cfh	GAGCCTGAGACCCAACTTCC	TGTGCAACGAAGGTAGTCCC	NM_009888.3
Csf1	AGTCTGTCTTCCACCTGCTG	TTCCACCTGTCTGTCCTCAT	NM_007778.4
Cxcl1	ACCCGCTCGCTTCTCTGT	AAGGGAGCTTCAGGGTCAAG	NM_008176.3
Cxcl2	CAGACTCCAGCCACACTTCA	TTCAGGGTCAAGGCAAACTT	NM_009140.2
Cxcl3	ATCCAGAGCTTGACGGTGAC	GGACTTGCCGCTCTTCAGTA	NM_203320.3
Cxcl10	GCCGTCATTTTCTGCCTCATCCT	CTCATTCTCACTGGCCCGTCATC	NM_021274.2
Cxcl12	CGCCAGAGCCAACGTCAAGC	TTCGGGTCAATGCACACTTG	NM_021704.3
Cxcr4	GAAGTGGGGTCTGGAGACTAT	TTGCCGACTATGCCAGTCAAG	NM_009911.3
Ddit4	CAAGGCAAGAGCTGCCATAG	CCGGTACTTAGCGTCAGGG	NM_029083.2
Fasl	CATGGAGTGGTCCTTAATGC	AAGTAGACCCACCCTGGAAG	NM_010177.4
Fgf2	CACCAGGCCACTTCAAGGA	GATGGATGCGCAGGAAGAA	NM_008006.2
Gapdh	CCAGCTCGTCCTGTAGACAA	GCCTTGACTGTGCCGTTGA	NM_008084.3
Hprt	GCTGACCTGCTGGATTACAT	TTGGGGCTGTACTGCTTAAC	NM_013556.2
Ifng	TTCTTCAGCAACAGCAAGGC	TCAGCAGCGACTCCTTTTCC	NM_008337.4
Il1a	CGGGTGACAGTATCAGCAAC	GACAAACTTCTGCCTGACGA	NM_010554.4
Il1b	CCCAACTGGTACATCAGCAC	TCTGCTCATTCACGAAAAGG	NM_008361.4
Il1rn	CCTCGGGATGGAAATCTGCTG	AGGCCTCGGCAGTACTATTGG	NM_031167.5
Il12a	GATGACATGGTGAAGACGGC	AGGCACAGGGTCATCATCAA	NM_008351.3
Ltb4r1	TGGCTGCAAACACTACATCTCCT	CACTGGCATACATGCTTATTCCAC	NM_008519.2
Mdk	GTCAATCACGCCTGTCCTCT	CAAGTATCAGGGTGGGGAGA	NM_010784.5
Mif	TGCCCAGAACCGCAACTACAGTAA	TCGCTACCGGTGGATAAACACAGA	NM_010798.3
Nos2	GGCCAGCCTGTGAGACCTTT	TTGGAAGTGAAGCGTTTCG	NM_010927.4
Osm	CTGTAAATGGGAACCTGTGG	TTCAAGAGGACTGGTTCAGC	NM_001013365.2
P2rx7	GGGTGGGGTGACGAAGTTAG	GGGTCCATCCACCCCTTTTT	NM_011027.4
Pcna	TAAAGAAGAGGAGGCGGTAA	TAAGTGTCCCATGTCAGCAA	NM_011045.2
Ptgs2	CAGACAACATAAACTGCGCCTT	GATACACCTCTCCACCAATGACC	NM_011198.4
Slpi	GCTGTGAGGGTATATGTGGGAAA	CGCCAATGTCAGGGATCAG	NM_011414.3
Tgfb1	GCTACCATGCCAACTTCTGT	CGTAGTAGACGATGGGCAGT	NM_011577.2
Tnfa	CCCACTCTGACCCCTTTACT	TTTGAGTCCTTGATGGTGGT	NM_013693.3
Tnfaip3	CGAGAGAGAACCCCAGAAGA	ATGCATGAGGCAGTTTCCA	NM_009397.3
Tnfsf10	CCTCTCGGAAAGGGCATTC	TCCTGCTCGATGACCAGCT	NM_009425.2
Vegfa	AGACACACCCACCCACATAC	CAGACCACACTGAAGCCTTT	NM_009505.4
Vegfb	CCTCTGAGCATGGAACTCAT	TTTGGTCTGCATTCACATTG	NM_011697.3

### Statistical analysis

All statistical analyses were performed using Prism Software (GraphPad). BMS scores and Ly6G^+^ neutrophil counts in spinal cord cross-sections were evaluated using a two-way, repeated measures ANOVA followed by Bonferroni’s multiple comparisons test. Comparison between two groups for flow cytometry, white matter sparing, and immunoblots was performed using an unpaired two-tailed Student’s *t* test. A one-way ANOVA followed by Tukey’s post hoc test was used for flow cytometry data comparing neutrophils from the blood and spinal cord of Syk^f/f^ and Syk^f/f^MRP8-Cre mice. To compare the proportion of animals with weight-supported stepping in the open field analysis (BMS), a chi-squared analysis was used followed by a one-sided Fisher’s exact test. Cytokine expression was evaluated using a two-way ANOVA followed by two-stage step-up method of Benjamini, Krieger, and Yekutieli correction for multiple comparisons with a false discovery rate (FDR) of 0.05. Only genes in which CT values could be determined for all three biological replicates were considered for statistical analysis (28/40 genes). Sex as a biological variable (SABV) was evaluated using a three-way, repeated measures ANOVA for BMS and a two-way ANOVA for all other assays. Significance was designated as *p*- and *q*-values < 0.05.

## Results

### Syk contributes to long-term neurologic deficits

**Fig. 1:**
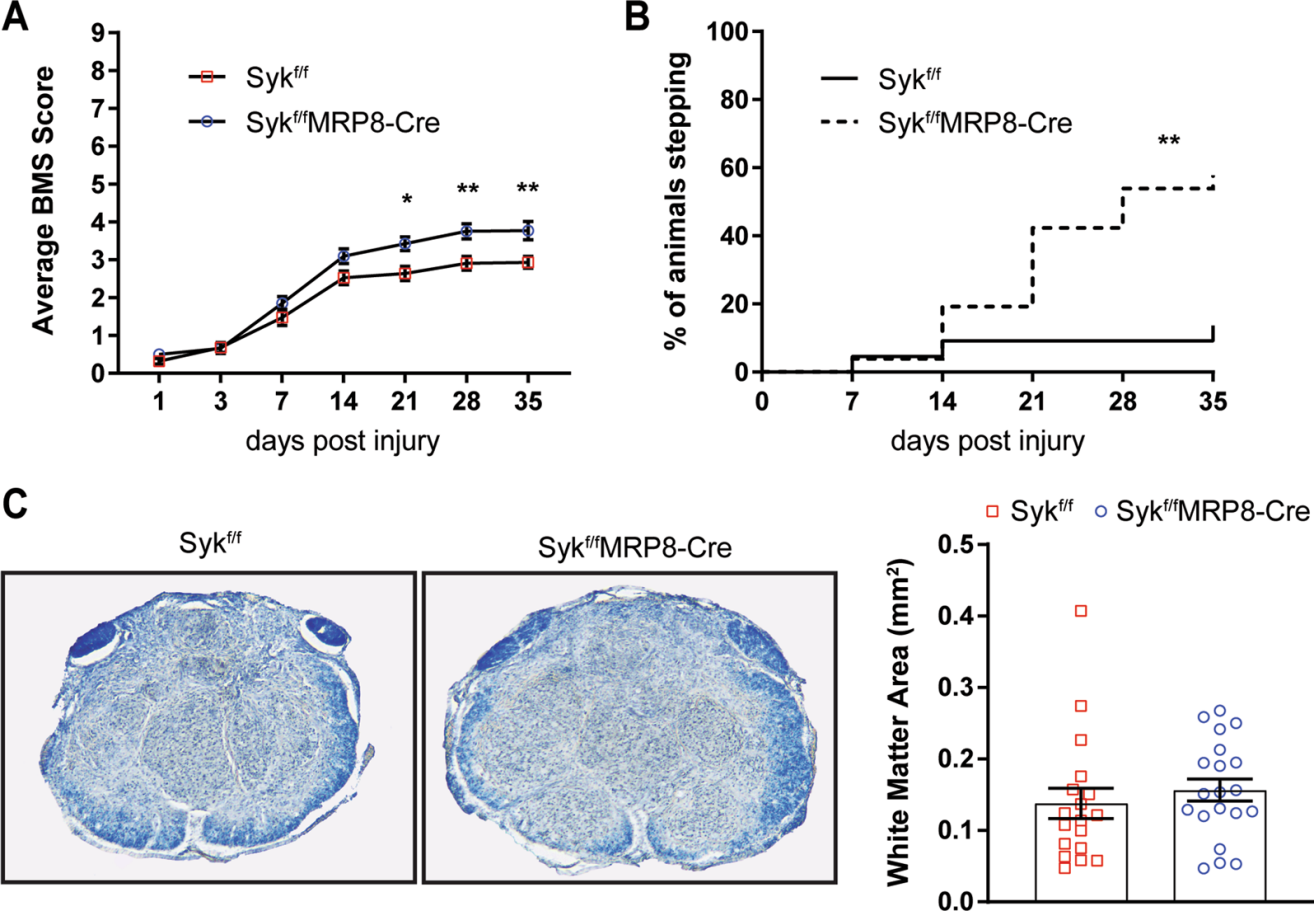
Neutrophil-specific deletion of Syk enhances long-term functional recovery, but not white matter sparing by 35 days post-SCI. **A** Greater BMS scores were observed in Syk^f/f^MRP8-Cre relative to Syk^f/f^ controls starting at 21 days post-SCI. N = 22 for Syk^f/f^ (13F/9M) and 26 for Syk^f/f^MRP8-Cre (16F/10M). Repeated measures two-way ANOVA with Bonferonni’s post hoc test. **B** Weight-supported stepping was improved in Syk^f/f^MRP8-Cre compared to Syk^f/f^ mice. *N* = 22 for Syk^f/f^ (13F/9M) and 26 for Syk^f/f^MRP8-Cre (16F/10M). *χ*^2^ analysis with Fischer’s exact test. **C** Representative images of the lesion epicenter, stained with eriochrome cyanine, in Syk^f/f^ and Syk^f/f^MRP8-Cre. No difference in white matter area was observed at 35 days post-SCI. *N* = 18 for Syk^f/f^ (13F/5M) and 20 for Syk^f/f^MRP8-Cre (14F/6M). Unpaired two-tailed Student’s *t* test. *p < 0.05, **p < 0.01. *SCI* spinal cord injury, *BMS* Basso mouse scale

To determine if Syk signaling in neutrophils contributes to long-term neurologic deficits after SCI, we examined functional recovery using the Basso Mouse Scale (BMS) following moderate thoracic contusion SCI at T9 in Syk^f/f^ and Syk^f/f^MRP8-Cre mice. Improved long-term recovery was observed in Syk^f/f^MRP8-Cre compared to Syk^f/f^ littermates starting at 21 days post-SCI and continuing through the end of the study (Fig. 1A; *p* = 0.01). No sex-specific effects were observed (*p* = 0.92). In a secondary analysis of the BMS data, approximately 60% of Syk^f/f^MRP8-Cre mice obtained weight-supported stepping (BMS ≥ 4), compared to 17% of Syk^f/f^ mice, by 35 days post-SCI (Fig. 1B; *p* < 0.01). Long-term white matter sparing after SCI was assessed between Syk^f/f^ and Syk^f/f^MRP8-Cre mice in spinal cord cross-sections stained with eriochrome cyanine. No differences were observed between the two genotypes in residual white matter area at the lesion epicenter at 35 days post-SCI (Fig. 1C; *p* = 0.24), indicating that improved functional recovery in Syk^f/f^MRP8-Cre mice was not due to greater sparing of white matter tissue at the SCI lesion site.

### Syk promotes neutrophil activation in the injured spinal cord

**Fig. 2:**
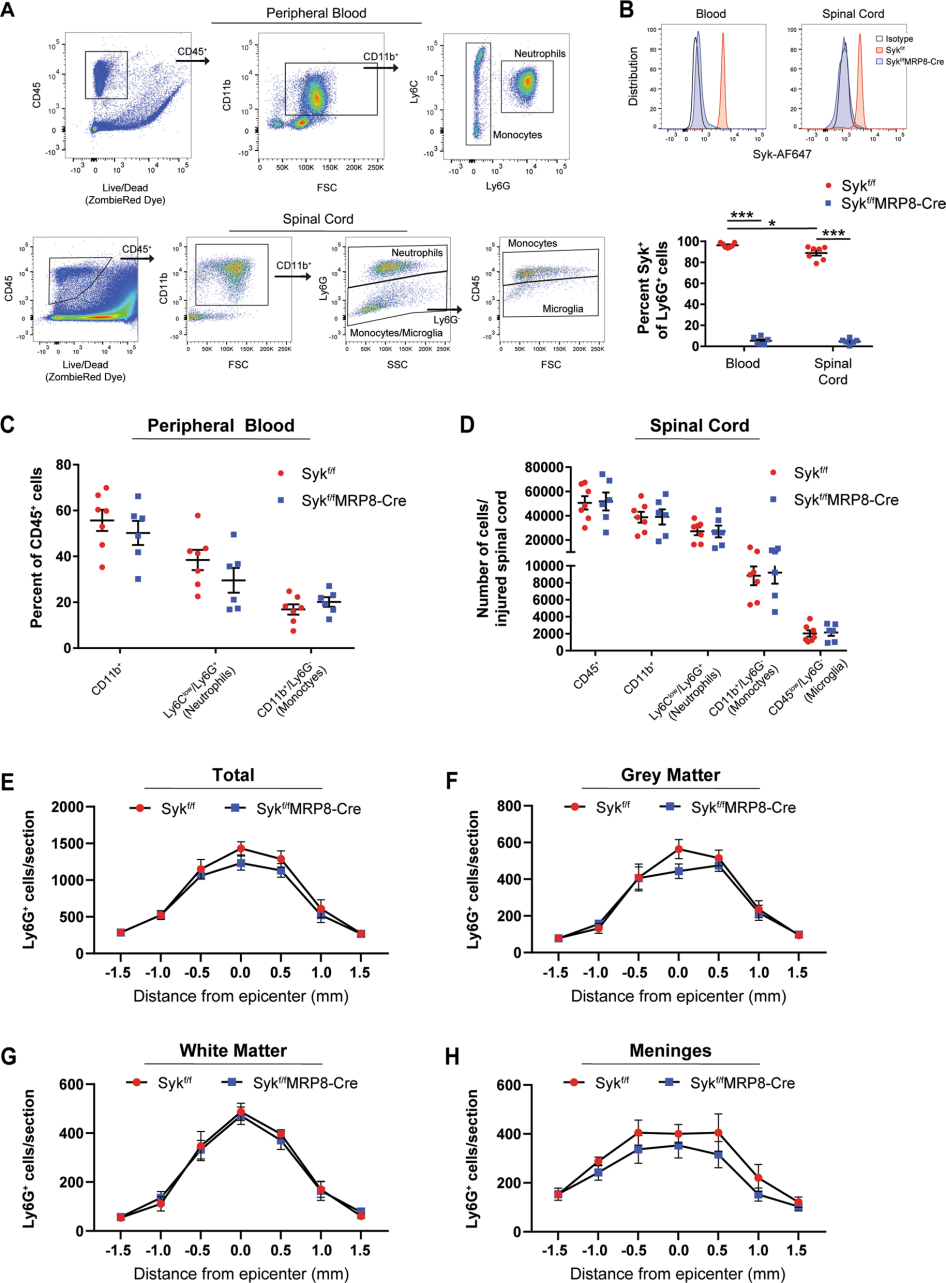
Syk-deficiency does not alter neutrophil accumulation in the acutely injured spinal cord. **A** Flow cytometry gating of leukocytes (CD45^+^), myeloid lineage cells (CD11b^+^), neutrophils (Ly6C^low^/Ly6G^+^), and monocytes (Ly6G^−^) in the blood and spinal cord at 1 days post-SCI. Microglia were identified in the spinal cord as CD11b^+^/Ly6G^−^/CD45^low^ cells. **B** Neutrophils from the blood and spinal cord of Syk^f/f^MRP8-Cre show robust Syk-deficiency relative to Syk^f/f^ controls at 1 days post-SCI. Two-way ANOVA with Tukey’s post hoc test. **C** Syk-deficiency does not alter the proportion of myeloid lineage cells in the peripheral blood compartment at 1 days post-SCI. **D** No differences were observed in the infiltration of leukocytes or myeloid lineage subtypes at 1 days post-SCI. *N* = 7 for Syk^f/f^ (4F/3M) and 6 for Syk^f/f^MRP8-Cre (3F/3M) for **B**–**D**. Unpaired two-tailed Student’s *t* test for **C**–**D**. **E** No differences were observed in the number of neutrophils (Ly6G^+^ cells) in sections spanning the lesion site in Syk^f/f^ and Syk^f/f^MRP8-Cre mice. **F**–**H** No differences were found in the accumulation of neutrophils in the grey matter, white matter, or meninges of Syk^f/f^ and Syk^f/f^MRP8-Cre mice. *N* = 5 for Syk^f/f^ (3F/2M) and 5 for Syk^f/f^MRP8-Cre (2F/3M) for E–H. Repeated measures two-way ANOVA with Bonferonni’s post hoc test. *p < 0.05, ***p < 0.001. *SCI* spinal cord injury

We performed flow cytometry analysis of injured spinal cords from Syk^f/f^ and Syk^f/f^MRP8-Cre mice at 1 days post-SCI to assess if Syk mediates neutrophil accumulation. Total leukocyte and myeloid cell populations were first identified with CD45 and CD11b antibodies, respectively. Myeloid cells were further separated into Ly6G^+^ (neutrophils) and Ly6G^−^ (monocyte) populations (Fig. 2A). In the spinal cord samples, monocyte and microglia populations were subdivided based on CD45^hi^ vs CD45^low^ levels, respectively. Intracellular staining for Syk was performed to determine the efficiency of the conditional knockout in circulating and infiltrated Ly6G^+^ neutrophils at 1 days post-SCI. In Syk^f/f^ mice, most Ly6G^+^ neutrophils (> 90%) showed positive immunolabeling for Syk, whereas few Ly6G^+^/Syk^+^ neutrophils (~ 5%) were detected in Syk^f/f^MRP8-Cre mice (Fig. 2B; *p* < 0.001). There was a modest decrease in Ly6G^+^/Syk^+^ neutrophils in the spinal cord compared to the blood of Syk^f/f^ mice (96.3% vs. 91.1%, *p* = 0.02). No differences were observed between Syk^f/f^ and Syk^f/f^MRP8-Cre mice in the proportion of circulating CD11b^+^ myeloid cells or myeloid subsets relative to the total CD45^+^ leukocyte population (Fig. 2C; *p* > 0.22 for all cell types). Furthermore, there were no differences in the number of accumulated CD45^+^ leukocytes or leukocyte subsets, including Ly6G^+^ neutrophils, in the acutely injured spinal cord (Fig. 2D; *p* > 0.39 for all cell types). We also performed semi-automated counting of Ly6G^+^ neutrophils in spinal cord cross-sections at every 500 µm across the SCI lesion (3 mm total) at 1 days post-SCI. No differences in the number of Ly6G^+^ neutrophils were observed across the SCI lesion (Fig. 2E; *p* = 0.19) or in the spinal cord grey matter (Fig. 2F; *p* = 0.34), white matter (Fig. 2G; *p* = 0.89), or spinal meninges (Fig. 2H; *p* = 0.33).

**Fig. 3:**
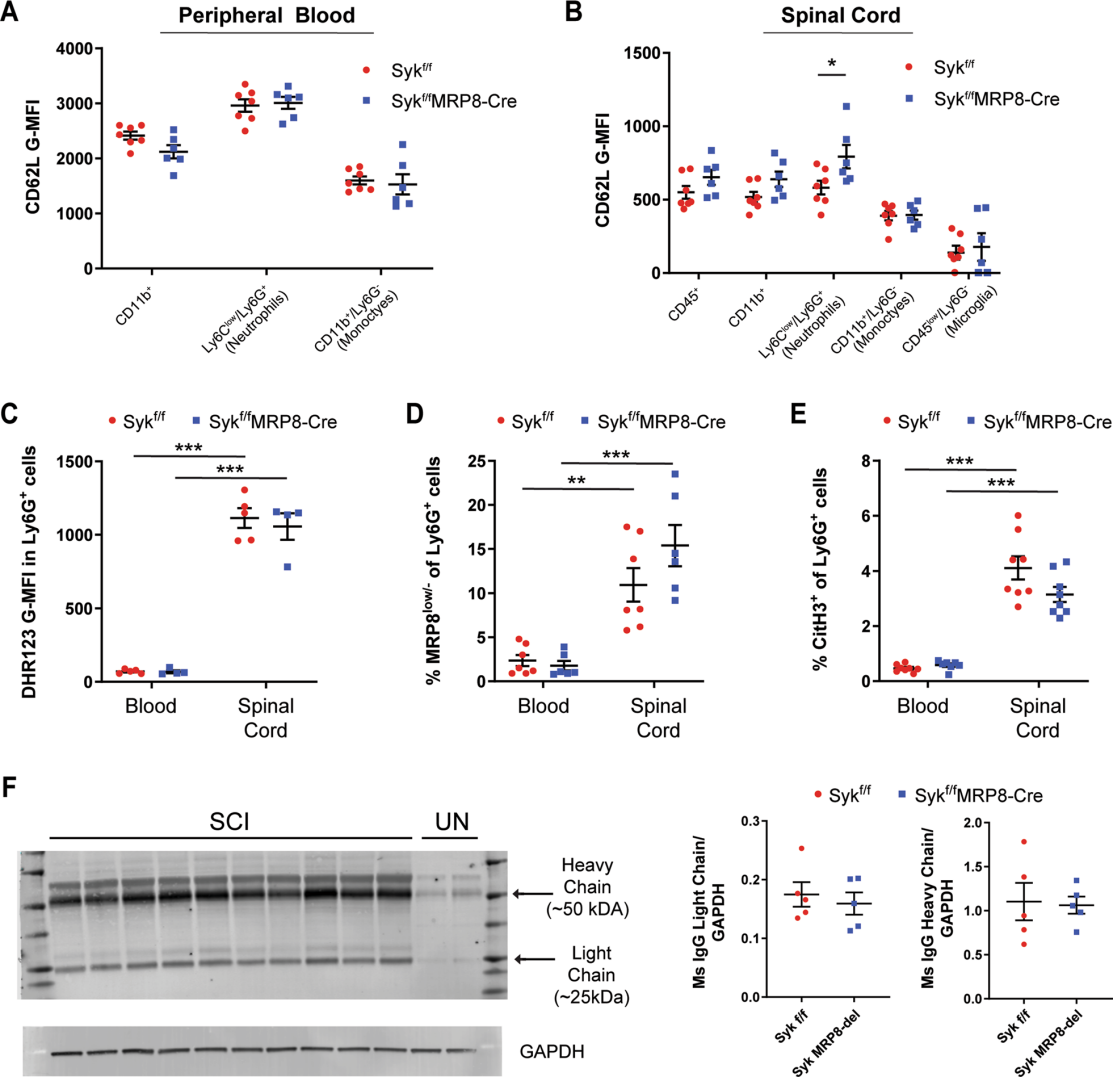
Syk reduces neutrophil activation, but does not mediate neutrophil effector functions, in the acutely injured spinal cord. **A** Similar CD62L levels (G-MFI) were detected between Syk^f/f^ and Syk^f/f^MRP8-Cre mice in the blood at 1 days post-SCI. **B** Syk-deficiency increased CD62L levels on neutrophils, but not other myeloid cell lineages, in the spinal cord of Syk^f/f^MRP8-Cre mice relative to Syk^f/f^ controls at 1 days post-SCI. N = 7 for Syk^f/f^ (4F/3M) and 6 for Syk^f/f^MRP8-Cre (3F/3M) for **A**-**B** Unpaired two-tailed Student’s *t* test for **A**–**B**. **C** Similar ROS levels, as measured by DHR123 G-MFI, were observed in blood or spinal cord neutrophils of Syk^f/f^ and Syk^f/f^MRP8-Cre mice at 1 days post-SCI. *N* = 5 for Syk^f/f^ (2F/3M) and 4 for Syk^f/f^MRP8-Cre (1F/3M). **D** No differences in the proportion of degranulated (MRP8^low/−^) neutrophils in blood or spinal cord were found between Syk^f/f^ and Syk^f/f^MRP8-Cre mice at 1 days post-SCI. *N* = 7 for Syk^f/f^ (3F/4M) and 6 for Syk^f/f^MRP8-Cre (2F/4M). **E** Syk-deficiency did not alter the proportion of neutrophils undergoing NETosis (CitH3^+^) in blood or spinal cord of Syk^f/f^ vs. Syk^f/f^MRP8-Cre mice at 1 days post-SCI. N = 8 for Syk^f/f^ (5F/3M) and 8 for Syk^f/f^MRP8-Cre (3F/5M). Two-way ANOVA with Tukey’s post hoc test for **C**–**E**. **F** Immunoblot for extravasated mouse IgG heavy and light chain subunits in the spinal cord at 1 days post-SCI. GAPDH was used as an internal control. No differences were observed in the levels of extravasated mouse IgG heavy or light chain subunits between Syk^f/f^ and Syk^f/f^MRP8-Cre mice. *N* = 5/genotype (2F/3M). Unpaired two-tailed Student’s *t* test for **F**. *p < 0.05. **p < 0.01, ***p < 0.001. *G-MFI* geometric mean fluorescent intensity, *ROS* reactive oxygen species, *SCI* spinal cord injury, *NET* neutrophil extracellular trap

To determine if the genetic loss of Syk affects neutrophil activation, we also performed flow cytometry analysis of L-selectin levels (CD62L G-MFI) on circulating and infiltrated leukocyte populations at 1 days post-SCI. Following activation, L-selectin undergoes proteolytic cleavage resulting in “shedding” of the ligand-binding ectodomain [49, 50]. Reduced L-selectin levels, therefore, are commonly used as an indicator of activation in leukocyte populations. We observed high, yet similar, levels of L-selectin on circulating CD11b^+^ myeloid cells, Ly6G^+^ neutrophils, and Ly6G^−^ monocyte populations from injured Syk^f/f^ and Syk^f/f^MRP8-Cre mice (Fig. 3A; *p* > 0.05 for all cell types). While lower L-selectin levels were observed on infiltrated leukocytes populations, Ly6G^+^ neutrophils from the injured spinal cords of Syk^f/f^MRP8-Cre mice had ~ 36% greater L-selectin levels relative to Syk^f/f^ mice indicative of reduced activation (Fig. 3B; *p* = 0.04). No sex-specific effects were observed (*p* = 0.84). No differences in L-selectin levels were observed for CD45^hi^/Ly6G^−^ monocytes or CD45^low^/Ly6G^−^ microglia in the acutely injured spinal cords of Syk^f/f^ and Syk^f/f^MRP8-Cre mice (*p* = 0.93 and 0.72, respectively).

### Syk does not facilitate common neutrophil effector functions acutely after SCI

Activated neutrophils can employ a wide range of responses depending on the encountered stimuli, although the specific effector functions utilized in the injured spinal cord have yet to be determined. To assess the effect of Syk-deletion on neutrophil effector functions acutely after SCI, we performed flow cytometry analysis of degranulation, ROS production, and NET formation on accumulated neutrophils acutely after SCI. There was a marked increase in all three effector functions in infiltrated neutrophils relative to their circulating counterparts at 1 days post-SCI (Fig. 3C–E; *p* < 0.01). No differences in the levels of ROS labeling (DHR123 G-MFI) were observed for Ly6G^+^ neutrophils in the blood or spinal cord of Syk^f/f^ vs. Syk^f/f^MRP8-Cre mice (Fig. 3C; *p* = 0.99 and 0.88, respectively). There were no differences observed in the proportion of Ly6G^+^ neutrophils with diminished MRP8 levels (Ly6G^+^/MRP8^low/−^), indicative of degranulation, in the blood or injured spinal cords of Syk^f/f^ relative to Syk^f/f^MRP8-Cre mice (Fig. 3D; *p* = 0.99 and 0.21, respectively). We also observed no differences in the proportion of Ly6G^+^ neutrophils undergoing NETosis (Ly6G^+^/CitH3^+^) in the blood or spinal cords of Syk^f/f^MRP8-Cre compared to Syk^f/f^ mice at 1 days post-SCI (Fig. 3E; *p* = 0.99 and 0.06, respectively).

Since there was minimal effect of Syk deficiency on the cytotoxic effector functions utilized by neutrophils in the acutely injured spinal cord, we sought to determine if there were any differences in acute secondary injury by assessing the accumulation of endogenous mouse IgG antibody, which can extravasate from the blood plasma into the spinal cord during blood-spinal cord barrier disruption [51]. We found no differences in accumulated mouse IgG light and heavy chain subunits, measured by immunoblotting, in the spinal cords of Syk^f/f^MRP8-Cre mice relative to Syk^f/f^ mice at 1 days post-SCI (Fig. 3F; *p* = 0.60 and 0.87, respectively) indicating that Syk in neutrophils does not contribute to acute blood-spinal cord barrier disruption.

### Syk augments neutrophil cell death in the acutely injured spinal cord

**Fig. 4:**
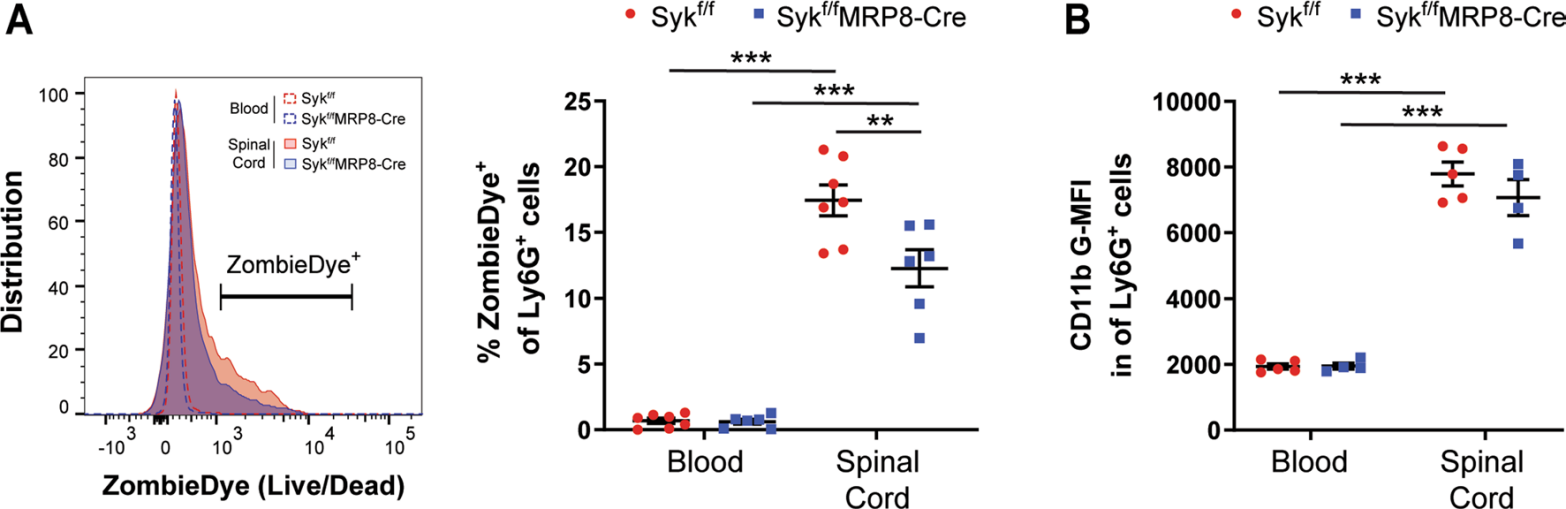
Syk augments neutrophil cell death in the acutely injured spinal cord independent of CD11b levels. **A** Representative flow cytometry histograms of ZombieDye levels in blood and spinal cord neutrophils. Syk deficiency reduced the proportion of intraspinal neutrophils labeled with ZombieDye at 1 days post-SCI. *N* = 7 for Syk^f/f^ (4F/3M) and 6 for Syk^f/f^MRP8-Cre (3F/3M). **B** No differences in CD11b levels (G-MFI) were found between Syk^f/f^ and Syk^f/f^MRP8-Cre mice at 1 days post-SCI. *N* = 5 for Syk^f/f^ (2F/3M) and 4 for Syk^f/f^MRP8-Cre (1F/3M). Two-way ANOVA with Tukey’s post hoc test for **A**, **B**. **p < 0.01, ***p < 0.001. *G-MFI* geometric mean fluorescent intensity, *SCI* spinal cord injury

While apoptosis has often been described as an anti-inflammatory cell fate pathway for activated neutrophils, studies have shown that dead neutrophils are not efficiently cleared in SCI [26, 27]. To determine if Syk contributes to neutrophil cell death in the injured spinal cord, we incubated dissociated spinal cord samples with a cell-impermeant, amine-fixable dye (ZombieDye) that brightly labels dead cells with compromised membrane integrity. While there was a substantial increase in Ly6G^+^/ZombieDye^+^ neutrophils in the injured spinal cord relative to the blood at 1 days post-SCI (Fig. 4A; *p* < 0.001), we observed a reduced proportion of Ly6G^+^/ZombieDye^+^ neutrophils in the injured spinal cords of Syk^f/f^MRP8-Cre relative to Syk^f/f^ mice at 1 days post-SCI (12.3% vs. 17.4%, respectively; *p* = 0.003). No sex-specific effects were observed (*p* = 0.88). β_2_ integrins, such as Mac-1 (CD11b/CD18), have been shown to contribute to neutrophil apoptosis [52], therefore, we also assessed the surface levels of CD11b on neutrophils by flow cytometry. While there was a marked increase in CD11b levels on Ly6G^+^ neutrophils in the spinal cord relative to the blood (*p* < 0.001), we found no differences in CD11b G-MFI on Ly6G^+^ neutrophils in the blood or spinal cord between Syk^f/f^ and Syk^f/f^MRP8-Cre mice (Fig. 4B; *p* = 0.99 and 0.41, respectively).

### Syk contributes to cytokine expression in neutrophils after SCI

**Fig. 5:**
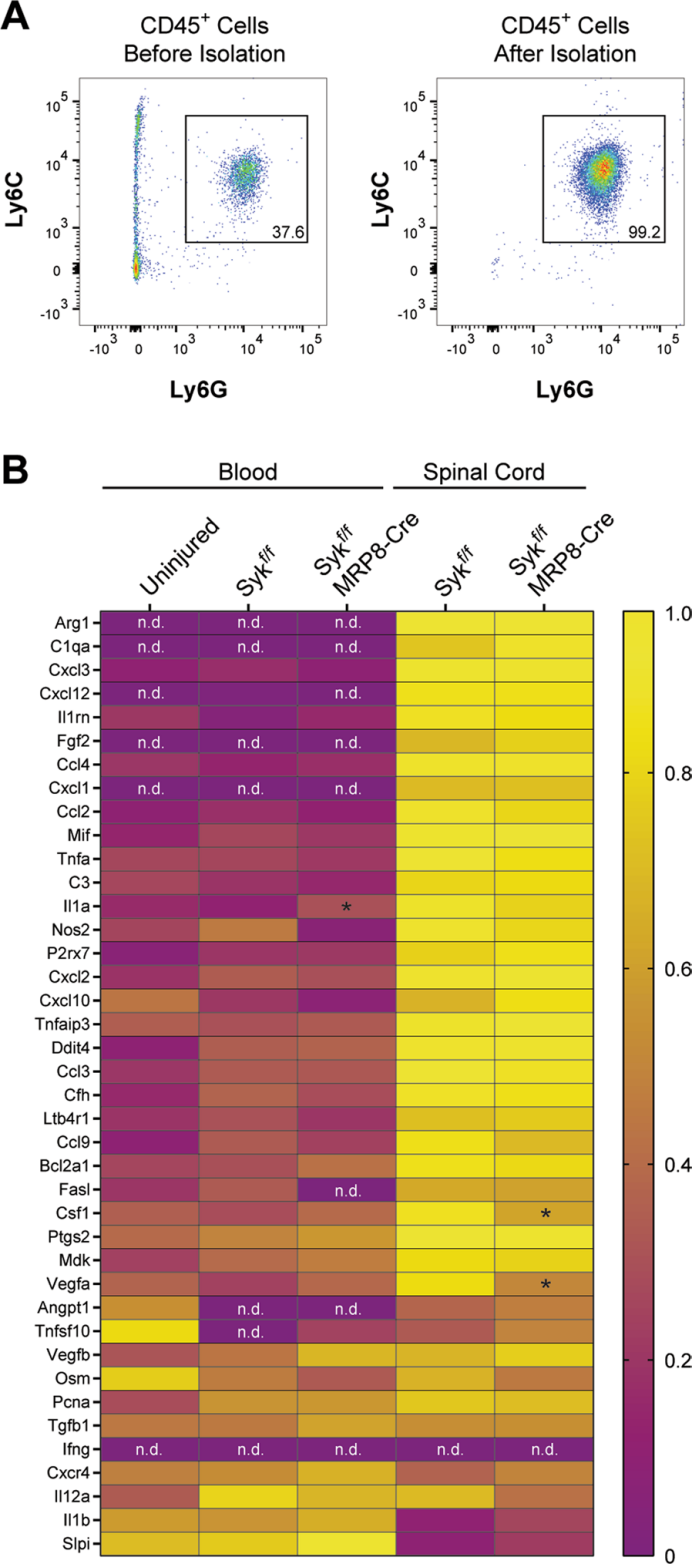
Syk deficiency partially attenuates the widespread upregulation of cytokines, chemokines, and other inflammatory mediators in infiltrated neutrophils in the acutely injured spinal cord. **A** Magnetic isolation enhanced neutrophil purity (> 99%) for qPCR analysis. **B** Heatmap of the average normalized CT value for 40 genes in neutrophils from the blood of uninjured Syk^f/f^, as well as the blood and spinal cords of Syk^f/f^ and Syk^f/f^MRP8-Cre mice at 1 days post-SCI. *Gapdh*, *Actb*, and *Hprt1* were used as internal controls. *N* = 3 for uninjured (2F/1M), 3 for Syk^f/f^ (1F/2M), and 3 for Syk^f/f^MRP8-Cre (0F/3M). *n.d.* not detected in at least 2/3 samples. Two-way ANOVA with Benjamini, Krieger, and Yekutieli multiple comparisons correction. *q < 0.05 relative to Syk^f/f^. *SCI* spinal cord injury)

We have previously shown that Syk signaling contributes to cytokine secretion in stimulated neutrophils in vitro [38], however, the role of Syk in the upregulation of cytokines and chemokines by neutrophils after SCI remains unknown. Furthermore, changes in the expression of cytokines and chemokines by neutrophils following infiltration into the acutely injured spinal cord have yet to be determined. To assess whether SCI and Syk signaling contribute to the expression of cytokines, chemokines, and other inflammatory mediators in neutrophils, we performed qPCR analysis of 40 genes (Table 1) using a gene array on mRNA from neutrophils isolated from the spinal cord and blood at 1 days post-SCI, as well as from the blood of uninjured Syk^f/f^ mice. Magnetic isolation was sufficient to achieve > 99% purity of Ly6G^+^ neutrophils from the blood and spinal cord (Fig. 5A) for mRNA isolation and analysis. We observed widespread changes in transcripts levels (ΔCT) for many of the genes in our panel (Fig. 5B). Transcripts for several genes (*Arg1*, *C1qa*, *Fgf2*, and *Cxcl1*) could not be detected in blood neutrophils, but were highly upregulated in neutrophils in the spinal cords of both Syk^f/f^ and Syk^f/f^MRP8-Cre mice. *Ifng* mRNA was not detected in either blood or spinal cord neutrophils.

Of the 40 genes in our panel, we performed statistical analysis on the 28 genes in which CT values could be detected for all samples (Table 2). Among these genes, we observed upregulation of *Ccl3* and *Cxcl2* in the blood of Syk^f/f^ mice acutely following SCI relative to uninjured controls. We found marked upregulation of several cytokines, chemokines, and inflammation-related genes (*Bcl2a1*, *Ccl3*, *Ccl4*, *Ccl9*, *Csf1, Cxcl2, Cxcl3, Cxcl10, Ddit4, Il1a, Il1rn, Mdk, Mif, P2rx7, Ptgs2, Tnfa, Tnfaip3*, and *Vegfa*) in infiltrated neutrophils relative to circulating neutrophils in both Syk^f/f^ and Syk^f/f^MRP8-Cre mice. Of the genes evaluated in our array, only *Slpi* was downregulated in infiltrated neutrophils relative to circulating neutrophils in both genotypes. When comparing between Syk^f/f^MRP8-Cre and Syk^f/f^ mice, we observed downregulation of *Csf1* (3.9-fold) and *Vegfa* (3.4-fold) in infiltrated neutrophils from Syk^f/f^MRP8-Cre at 1 days post-SCI (*q* = 0.009 and 0.02, respectively). *Il1a* was upregulated 3.1-fold in the blood of Syk^f/f^MRP8-Cre mice relative to Syk^f/f^ controls at 1 days post-SCI (*q* = 0.03). Our findings indicate that Syk signaling contributes to the expression of specific cytokines in neutrophils after SCI.

**Table 2:** Gene expression (ΔCT) in blood and spinal cord neutrophils

Gene symbol	Syk^f/f^ Δ*CT* values			Syk^f/f^MRP8-Cre Δ*CT* values		FDR *q* value
	UN	Blood	Spinal Cord	Blood	Spinal cord	
Bcl2a1	9.22	8.86	4.18	7.80	4.53	^b^*q* < 0.001
C3	1.52	1.72	0.01	1.83	− 0.05	
Ccl3	10.11	8.43	1.14	8.53	1.82	^b^*q* < 0.001, ^d^*q* = 0.026
Ccl4	7.11	7.61	1.68	7.3	1.75	^b^*q* < 0.001
Ccl9	10.55	9.37	6.84	9.82	7.58	^b^*q* < 0.01
Cox2	8.98	8.36	5.67	7.84	5.49	^b^*q* < 0.01
Csf1	9.93	10.45	5.78	9.58	7.76	^b^*q* < 0.05, ^a^*q* = 0.005
Cxcl2	10.00	8.31	1.82	8.83	2.30	^b^*q* < 0.001, ^d^*q* = 0.025
Cxcl3	11.03	10.18	− 0.23	11.12	0.25	^b^*q* < 0.001
Cxcl10	8.38	9.49	7.27	10.08	6.47	^b^*q* < 0.01
Cxcr4	0.81	0.63	1.19	0.16	0.73	
Ddit4	8.45	7.38	4.69	7.27	4.98	^b^*q* < 0.01
Il1a	14.38	14.83	8.14	13.18	8.95	^b^*q* < 0.001, ^c^*q* = 0.013
Il1b	− 1.50	− 1.43	− 0.12	− 1.70	− 0.56	
Il1rn	2.71	3.41	− 0.10	2.94	0.07	^b^*q* < 0.001
Il12a	10.78	8.85	9.26	9.34	10.41	
Ltb4r1	2.79	2.50	1.43	2.77	1.34	
Mdk	12.69	11.70	9.06	11.25	9.28	^b^*q* < 0.01
Mif	4.05	3.59	0.95	3.80	1.07	^b^*q* < 0.001
Osm	9.72	10.40	9.95	10.70	10.43	
P2rx7	12.61	12.22	10.43	12.18	10.23	^b^*q* < 0.05
Pcna	6.71	5.99	5.49	5.96	5.61	
Slpi	− 0.77	− 0.97	1.19	− 1.50	0.78	^b^*q* < 0.01
Tgfb1	4.26	4.24	3.99	3.74	3.97	
Tnfa	8.43	8.44	5.36	8.68	5.76	^b^*q* < 0.001
Tnfaip3	6.89	7.08	4.33	6.93	4.24	^b^*q* < 0.001
Vegfa	11.77	12.48	9.29	11.68	11.04	^b^*q* < 0.01, ^a^*q* = 0.021
Vegfb	8.21	7.99	7.58	7.57	7.42	

Values represent average ΔCT for each group. For each sample, CT values were normalized to the average of Gapdh, Actb, and Hprt1. *N* = 3/group. Two-way ANOVA with Benjamini, Krieger, and Yekutieli multiple comparisons correction

*FDR* false discovery rate

^a^*q* < 0.05 for spinal cord neutrophils between genotypes

^b^*q* < 0.05 for blood neutrophils vs. spinal cord neutrophils (Syk ^f/f^ and Syk^f/f^MRP8Cre mice)

^c^*q* < 0.05 for blood neutrophils between genotypes

^d^*q* < 0.05 for UN blood neutrophils vs. SCI blood neutrophils (Syk^f/f^ mice)

## Discussion

Tyrosine kinases, including Syk, help coordinate the complex intracellular signaling pathways downstream of immunoreceptors in neutrophils to enable appropriate cellular responses to the extracellular cues sensed at injury and infection sites [32, 33]. However, relatively little is known about the functional responses of neutrophils in the injured spinal cord or the role of Syk in mediating pathogenic neutrophil activities after SCI. Here we show that Syk augments neutrophil activation, cell death, and chemokine expression, but does not mediate the development of neutrophil effector functions within the acutely injured spinal cord. We demonstrate the neutrophil-specific deletion of Syk improves long-term functional recovery, but does not alter long-term white matter sparing. Our findings indicate that Syk mediates pathogenic neutrophil activities in the injured spinal cord that exacerbate long-term neurologic deficits through mechanisms independent of long-term white matter tissue loss.

Reducing neutrophil accumulation into the acutely injured spinal cord, by targeting adhesion receptors or inflammatory mediators involved in the recruitment of neutrophils, has predominantly led to improved tissue sparing and long-term functional recovery [4, 7–10, 46]. In our study, we demonstrate that Syk signaling does not affect neutrophil accumulation or distribution at 1 days post-SCI, which is the time point when neutrophil numbers have been previously reported to peak in murine models of SCI [4, 7, 12, 13]. Our results are consistent with studies in other models of inflammation that have shown that Syk does not substantially contribute to neutrophil migration or recruitment [38, 53]. However, we still observed improved long-term recovery in Syk^f/f^MRP8-Cre mice, demonstrating that Syk signaling in neutrophils mediates neurologic deficits after contusive SCI. To the best of our knowledge, this is the first report of improved long-term recovery after SCI by disrupting neutrophil function independent of recruitment. Interestingly, the improved behavioral outcomes did not coincide with long-term white matter sparing in our study. Furthermore, we did not observe any differences in blood-spinal cord barrier disruption in Syk-deficient mice, as measured by the extravasation of endogenous mouse antibody at 1 days post-SCI. These results indicate that Syk does not mediate neutrophil functions involved in secondary tissue damage, but could contribute to other neutrophil & inflammatory processes that impair regeneration, neural circuit reorganization, or neuronal activity. In addition, Syk signaling may contribute to neutrophil function outside of the spinal cord and promote systemic pathological conditions, such as the development of non-alcoholic steatohepatitis [54], that could exacerbate neurologic deficits.

Recent studies have shown that neutrophils acquire distinct functional phenotypes in different tissue environments [55], however, the pathogenic activities of neutrophils within the injured spinal cord have yet to be determined. We found that Syk-deficient neutrophils acquire lower levels of activation in the acutely injured spinal cord, as indicated by greater CD62L (L-selectin) levels at 1 days post-SCI. These results are consistent with our past study that demonstrated reduced L-selectin shedding following stimulation of Syk-deficient neutrophils in vitro [38]. We also evaluated the major effector functions utilized by activated neutrophils in the injured spinal cord. While we observed substantial increases in ROS production, degranulation, and NETosis in infiltrated neutrophils relative to their circulating counterparts, we did not see any attenuation of neutrophil effector functions with Syk deficiency. These findings differ from our previous results, which demonstrated that ROS production or degranulation are augmented by Syk signaling in response to microbial stimuli in vitro [38]. One limitation in our current study is that we were only able to measure intracellular ROS production in infiltrated neutrophils ex vivo. We cannot exclude the possibility of altered extracellular release of ROS in Syk-deficient neutrophils or artifacts introduced by the tissue dissociation and the brief culture of infiltrated neutrophils ex vivo. Regardless, our results suggest that Syk does not substantially impact neutrophil effector functions in the acutely injured spinal cord. These findings, combined with the similar infiltration of Syk-deficient neutrophils into the injured spinal cord, might explain why no differences were observed in acute blood-spinal cord barrier disruption or long-term white matter sparing. Future work is needed to determine the role of Syk signaling in neutrophils that are present in the injured spinal cord after 1 days post-SCI.

While neutrophils typically express lower levels of cytokines relative to other immune cell types, their large numbers can still influence the cytokine and chemokine profiles within the injury site and profoundly shape the course of inflammation [28, 29]. We previously found that Syk can potentiate cytokine secretion by neutrophils following stimulation with *S. aureus* or *E. coli* in vitro [38]. Cytokine expression by neutrophils in the injured spinal cord, however, has yet to be described. In this study, we developed a 40-gene panel to broadly screen for cytokines, chemokines, and other inflammatory mediators that have been previously shown to be expressed by neutrophils in other models of inflammation or infection [28–31]. We found that activated neutrophils in the spinal cord of both genotypes markedly upregulated many of the genes assessed in our panel relative to their circulating counterparts. Interestingly, we found that neutrophils in the spinal cord downregulate *SLPI*, a serine protease inhibitor that has been previously shown to promote recovery after SCI [56]. To the best of our knowledge, this is the first study to broadly assess the expression of cytokine and chemokine genes in neutrophils isolated from the acutely injured spinal cord. We observed that Syk-deficient neutrophils in the injured spinal cord have lower *Csf1* transcript levels relative to control Syk^f/f^ mice at 1 days post-SCI. Csf1 is a prominent chemokine involved in the proliferation and polarization of macrophages and microglia with detrimental effects in SCI and peripheral nerve injury [57, 58]. Our findings suggest that Syk signaling can facilitate the production of chemokines that may impact subsequent inflammation or tissue repair.

Neutrophil apoptosis is typically considered an inflammation resolving process that can induce reparative macrophage phenotypes upon phagocytosis by the macrophages [22–24]. However, the capacity of macrophages to clear dead neutrophils is impaired by myelin phagocytosis [26, 27], which may lead to the release of cytotoxic neutrophil contents into the spinal cord parenchyma. Neutrophil apoptosis has been shown to be accelerated by the engagement of β_2_ integrins, including CD11b/CD18 (Mac-1) and CD11d/CD18, while in the presence of pro-inflammatory cytokines including TNF-α [52]. Syk mediates intracellular signaling downstream of Mac-1 and CD11d/CD18 [53], though the role of Syk in neutrophil cell death has not been determined. We found similarly upregulated levels of CD11b on infiltrated neutrophils in both genotypes, however, Syk^f/f^MRP8-Cre mice showed reduced cell death relative to Syk^f/f^ mice. These findings suggest that Syk signaling may participate in integrin-mediated acceleration of neutrophil cell death in the injured spinal cord, which could directly impact neurons and glia in the spinal cord or indirectly influence other inflammatory processes such as macrophage polarization. Abrogated neutrophil apoptosis may also contribute to the benefit observed with CD11d/CD18 blocking antibody treatment after SCI [8].

## Conclusions

Collectively, our results provide the first extensive characterization of neutrophil functional responses in the acutely injured cord and establish Syk signaling as a critical mediator of long-term neurologic deficits after SCI. We demonstrate, to the best of our knowledge, the first evidence of NET formation in murine SCI. This work represents a key step in understanding how neutrophils respond to the inflammatory environment of the injured spinal cord and identifies neutrophil cytokine expression and cell death as potential pathogenic events in the acutely injured spinal cord. Future studies are needed to determine how Syk signaling in neutrophils, as well as neutrophil cell death and cytokine expression, contribute to neurologic deficits independent of long-term tissue sparing.

## Data Availability

The datasets used and/or analysed during the current study are available from the corresponding author on reasonable request.

## Abbreviations

BMS: Basso Mouse Scale
Syk: Spleen tyrosine kinase
SCI: Spinal cord injury
G-MFI: Geometric mean fluorescent intensity
NETs: Neutrophil extracellular traps
ROS: Reactive oxygen species
RNS: Reactive nitrogen species
ITAMs: Immunoreceptor tyrosine activation motifs
NDS: Normal donkey serum
TBS: Tris-buffered saline
